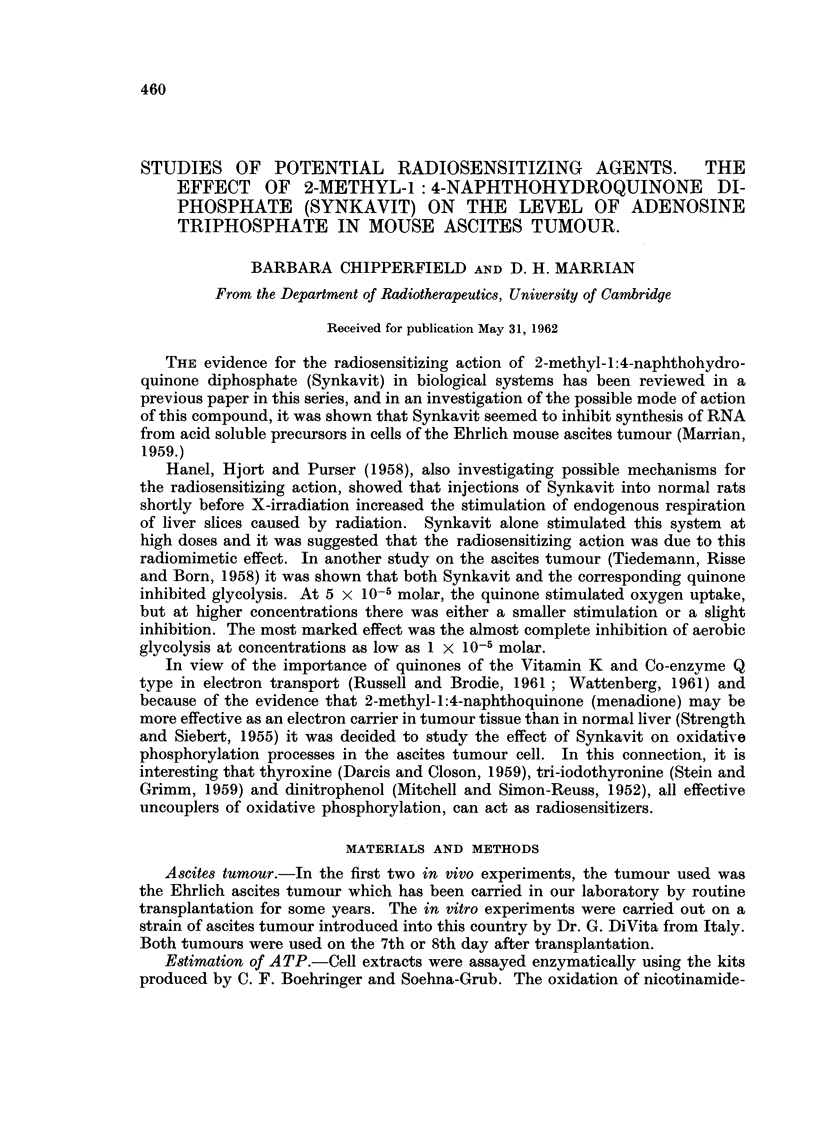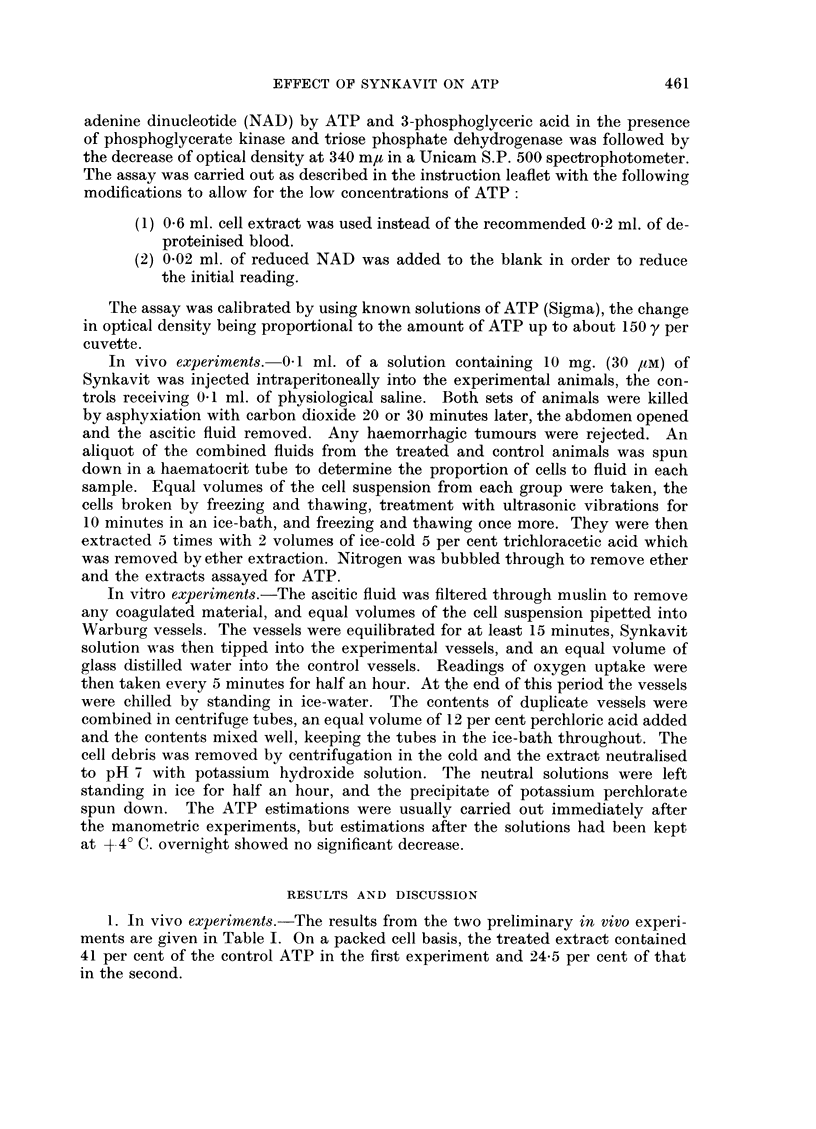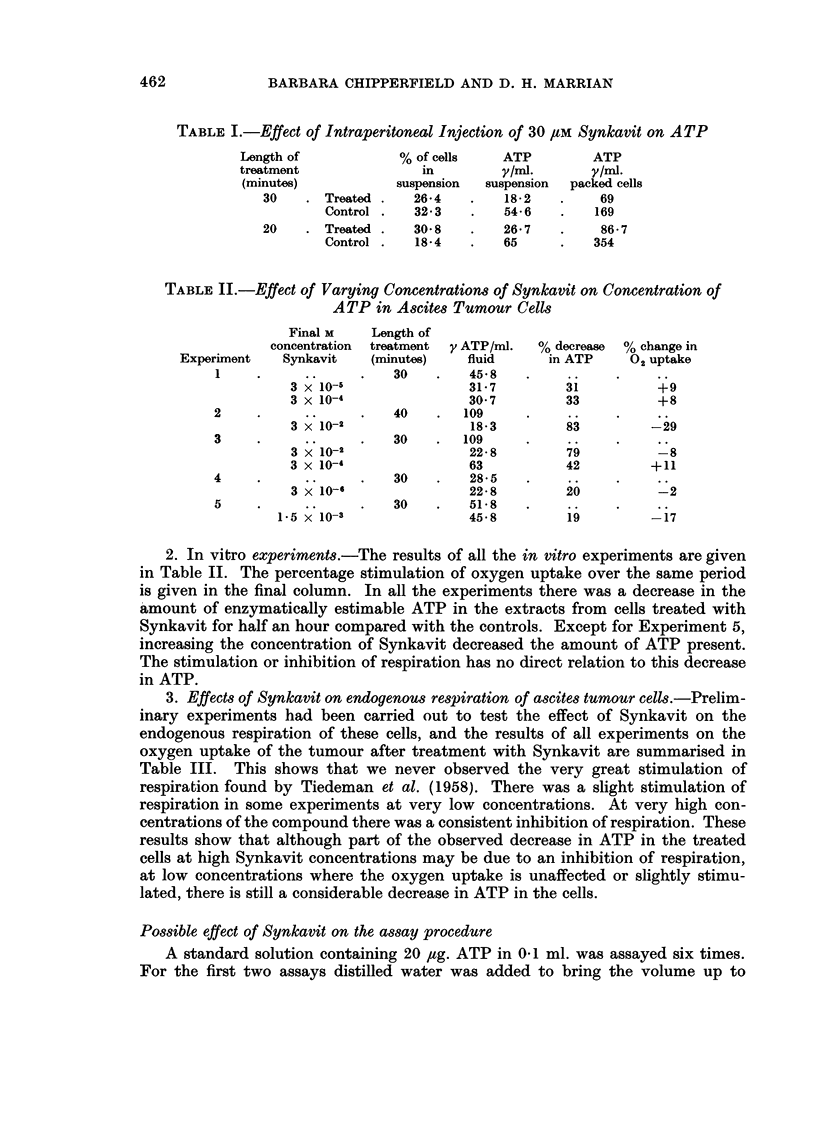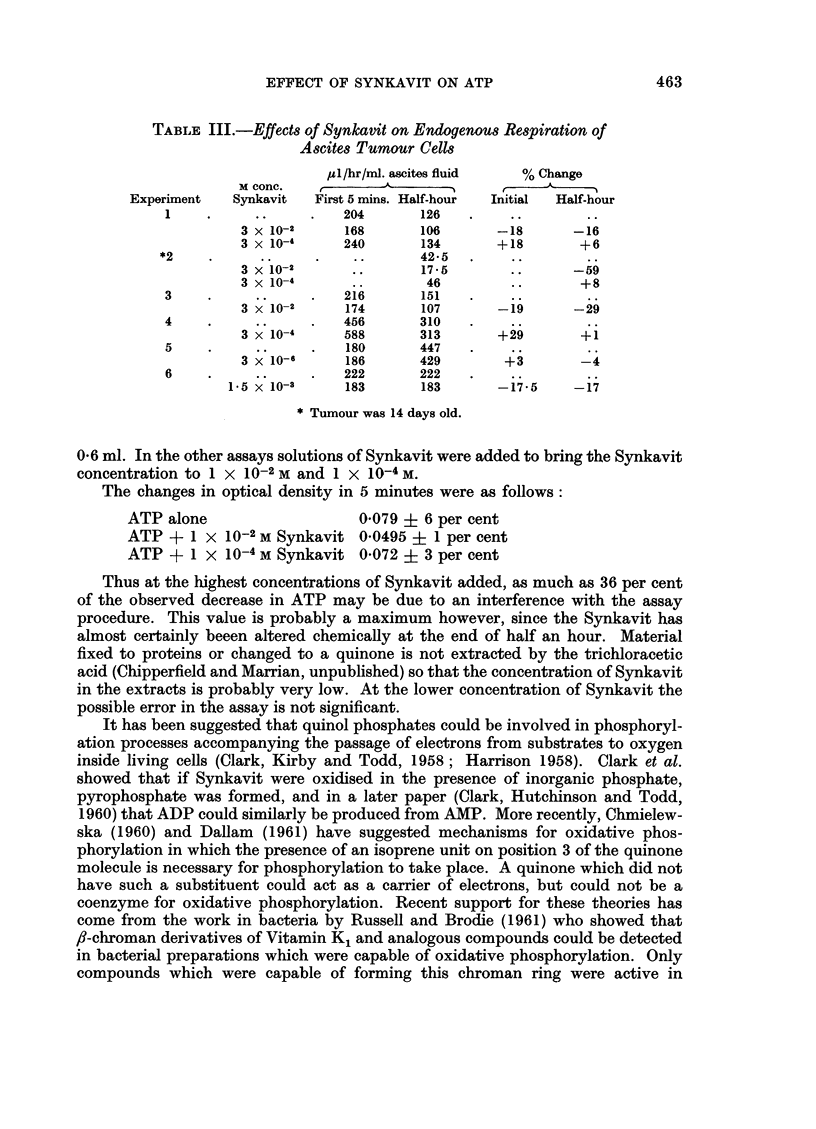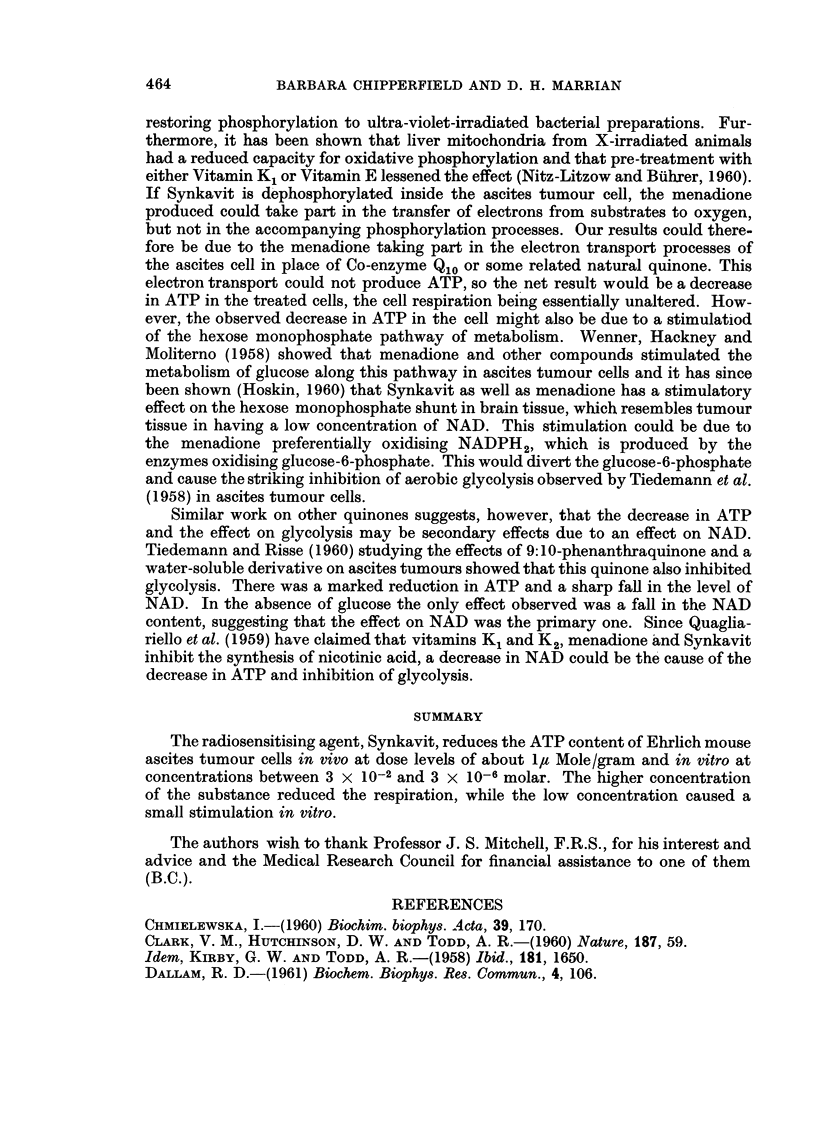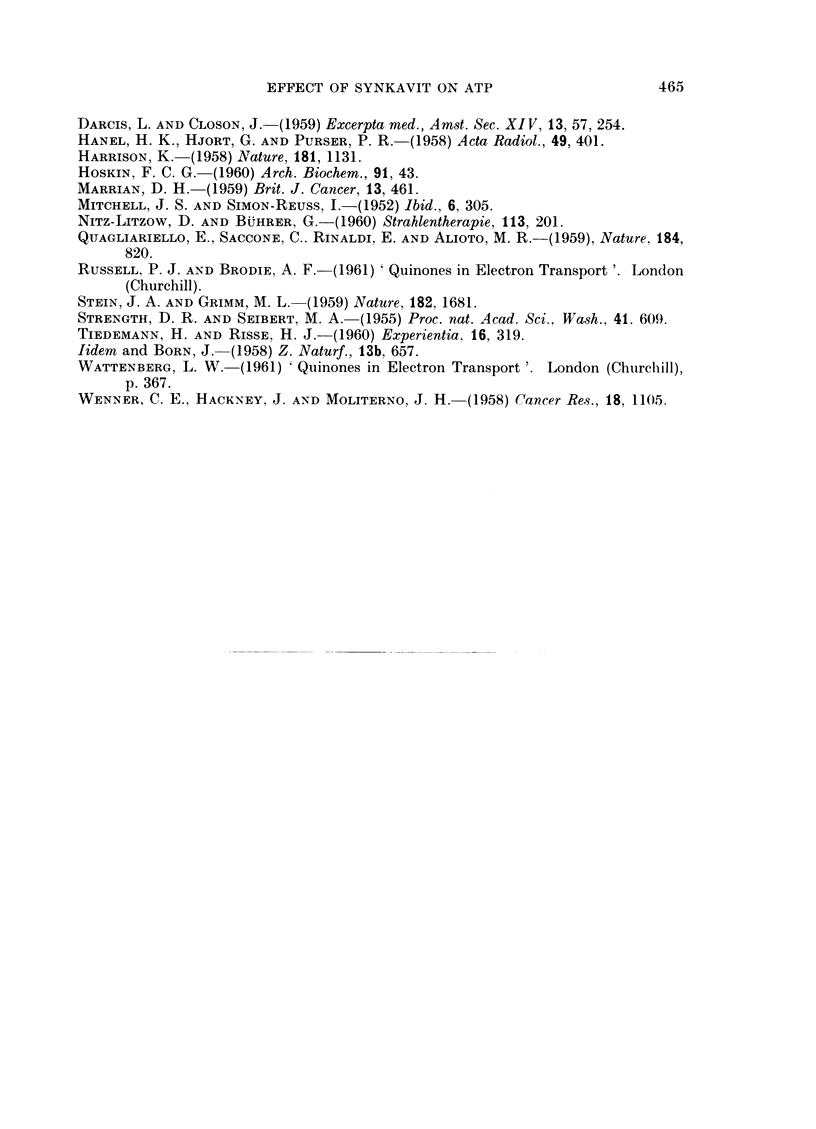# Studies of Potential Radiosensitizing Agents. The Effect of 2-Methyl-1: 4-Naphthohydroquinone Diphosphate (Synkavit) on the level of Adenosine Triphosphate in Mouse Ascites Tumour

**DOI:** 10.1038/bjc.1962.51

**Published:** 1962-09

**Authors:** Barbara Chipperfield, D. H. Marrian


### STUDIES OF POTENTIAL RADIOSENSITIZING AGENTS. THE EFFECT OF 2-METHYL-1 : 4-NAPHTHOHYDROQUINONE DIPHOSPHATE (SYNKAVIT) ON THE LEVEL OF ADENOSINE TRIPHOSPHATE IN MOUSE ASCITES TUMOUR.

BARBARA CHIPPERFIELD AND D. H. MARRIAN

*From the Department of Radiotherapeutics, University of Cambridge*

Received for publication May 31, 1962

THE evidence for the radiosensitizing action of 2-methyl-1:4-naphthohydroquinone diphosphate (Synkavit) in biological systems has been reviewed in a previous paper in this series, and in an investigation of the possible mode of action of this compound, it was shown that Synkavit seemed to inhibit synthesis of RNA from acid soluble precursors in cells of the Ehrlich mouse ascites tumour (Marrian, 1959.)

Hanel, Hjort and Purser (1958), also investigating possible mechanisms for the radiosensitizing action, showed that injections of Synkavit into normal rats shortly before X-irradiation increased the stimulation of endogenous respiration of liver slices caused by radiation. Synkavit alone stimulated this system at high doses and it was suggested that the radiosensitizing action was due to this radiomimetic effect. In another study on the ascites tumour (Tiedemann, Risse and Born, 1958) it was shown that both Synkavit and the corresponding quinone inhibited glycolysis. At $5 \times 10^{-5}$ molar, the quinone stimulated oxygen uptake, but at higher concentrations there was either a smaller stimulation or a slight inhibition. The most marked effect was the almost complete inhibition of aerobic glycolysis at concentrations as low as $1 \times 10^{-5}$ molar.

In view of the importance of quinones of the Vitamin K and Co-enzyme Q type in electron transport (Russell and Brodie, 1961 ; Wattenberg, 1961) and because of the evidence that 2-methyl-1:4-naphthoquinone (menadione) may be more effective as an electron carrier in tumour tissue than in normal liver (Strength and Siebert, 1955) it was decided to study the effect of Synkavit on oxidative phosphorylation processes in the ascites tumour cell. In this connection, it is interesting that thyroxine (Darcis and Closon, 1959), tri-iodothyronine (Stein and Grimm, 1959) and dinitrophenol (Mitchell and Simon-Reuss, 1952), all effective uncouplers of oxidative phosphorylation, can act as radiosensitizers.

### MATERIALS AND METHODS

*Ascites tumour.*—In the first two *in vivo* experiments, the tumour used was the Ehrlich ascites tumour which has been carried in our laboratory by routine transplantation for some years. The *in vitro* experiments were carried out on a strain of ascites tumour introduced into this country by Dr. G. DiVita from Italy. Both tumours were used on the 7th or 8th day after transplantation.

*Estimation of ATP.*—Cell extracts were assayed enzymatically using the kits produced by C. F. Boehringer and Soehna-Grub. The oxidation of nicotinamideadenine dinucleotide (NAD) by ATP and 3-phosphoglyceric acid in the presence of phosphoglycerate kinase and triose phosphate dehydrogenase was followed by the decrease of optical density at 340 m$\mu$ in a Unicam S.P. 500 spectrophotometer. The assay was carried out as described in the instruction leaflet with the following modifications to allow for the low concentrations of ATP :

(1) 0·6 ml. cell extract was used instead of the recommended 0·2 ml. of deproteinised blood.
(2) 0·02 ml. of reduced NAD was added to the blank in order to reduce the initial reading.

The assay was calibrated by using known solutions of ATP (Sigma), the change in optical density being proportional to the amount of ATP up to about 150 $\gamma$ per cuvette.

In vivo *experiments.*—0·1 ml. of a solution containing 10 mg. (30 $\mu$M) of Synkavit was injected intraperitoneally into the experimental animals, the controls receiving 0·1 ml. of physiological saline. Both sets of animals were killed by asphyxiation with carbon dioxide 20 or 30 minutes later, the abdomen opened and the ascitic fluid removed. Any haemorrhagic tumours were rejected. An aliquot of the combined fluids from the treated and control animals was spun down in a haematocrit tube to determine the proportion of cells to fluid in each sample. Equal volumes of the cell suspension from each group were taken, the cells broken by freezing and thawing, treatment with ultrasonic vibrations for 10 minutes in an ice-bath, and freezing and thawing once more. They were then extracted 5 times with 2 volumes of ice-cold 5 per cent trichloracetic acid which was removed by ether extraction. Nitrogen was bubbled through to remove ether and the extracts assayed for ATP.

In vitro *experiments.*—The ascitic fluid was filtered through muslin to remove any coagulated material, and equal volumes of the cell suspension pipetted into Warburg vessels. The vessels were equilibrated for at least 15 minutes, Synkavit solution was then tipped into the experimental vessels, and an equal volume of glass distilled water into the control vessels. Readings of oxygen uptake were then taken every 5 minutes for half an hour. At the end of this period the vessels were chilled by standing in ice-water. The contents of duplicate vessels were combined in centrifuge tubes, an equal volume of 12 per cent perchloric acid added and the contents mixed well, keeping the tubes in the ice-bath throughout. The cell debris was removed by centrifugation in the cold and the extract neutralised to pH 7 with potassium hydroxide solution. The neutral solutions were left standing in ice for half an hour, and the precipitate of potassium perchlorate spun down. The ATP estimations were usually carried out immediately after the manometric experiments, but estimations after the solutions had been kept at $+4^\circ$ C. overnight showed no significant decrease.

### RESULTS AND DISCUSSION

1. In vivo *experiments.*—The results from the two preliminary *in vivo* experiments are given in Table I. On a packed cell basis, the treated extract contained 41 per cent of the control ATP in the first experiment and 24·5 per cent of that in the second.

TABLE I.—*Effect of Intraperitoneal Injection of* 30 $\mu$M *Synkavit on ATP*

| Length of treatment (minutes) | | % of cells in suspension | ATP $\gamma$/ml. suspension | ATP $\gamma$/ml. packed cells |
|---|---|---|---|---|
| 30 | Treated | 26·4 | 18·2 | 69 |
| | Control | 32·3 | 54·6 | 169 |
| 20 | Treated | 30·8 | 26·7 | 86·7 |
| | Control | 18·4 | 65 | 354 |

TABLE II.—*Effect of Varying Concentrations of Synkavit on Concentration of ATP in Ascites Tumour Cells*

| Experiment | Final M concentration Synkavit | Length of treatment (minutes) | $\gamma$ ATP/ml. fluid | % decrease in ATP | % change in $O_2$ uptake |
|---|---|---|---|---|---|
| 1 | .. | 30 | 45·8 | .. | .. |
| | $3 \times 10^{-5}$ | | 31·7 | 31 | +9 |
| | $3 \times 10^{-4}$ | | 30·7 | 33 | +8 |
| 2 | .. | 40 | 109 | .. | .. |
| | $3 \times 10^{-2}$ | | 18·3 | 83 | −29 |
| 3 | .. | 30 | 109 | .. | .. |
| | $3 \times 10^{-2}$ | | 22·8 | 79 | −8 |
| | $3 \times 10^{-4}$ | | 63 | 42 | +11 |
| 4 | .. | 30 | 28·5 | .. | .. |
| | $3 \times 10^{-6}$ | | 22·8 | 20 | −2 |
| 5 | .. | 30 | 51·8 | .. | .. |
| | $1{\cdot}5 \times 10^{-3}$ | | 45·8 | 19 | −17 |

2. In vitro *experiments*.—The results of all the *in vitro* experiments are given in Table II. The percentage stimulation of oxygen uptake over the same period is given in the final column. In all the experiments there was a decrease in the amount of enzymatically estimable ATP in the extracts from cells treated with Synkavit for half an hour compared with the controls. Except for Experiment 5, increasing the concentration of Synkavit decreased the amount of ATP present. The stimulation or inhibition of respiration has no direct relation to this decrease in ATP.

3. *Effects of Synkavit on endogenous respiration of ascites tumour cells*.—Preliminary experiments had been carried out to test the effect of Synkavit on the endogenous respiration of these cells, and the results of all experiments on the oxygen uptake of the tumour after treatment with Synkavit are summarised in Table III. This shows that we never observed the very great stimulation of respiration found by Tiedeman *et al.* (1958). There was a slight stimulation of respiration in some experiments at very low concentrations. At very high concentrations of the compound there was a consistent inhibition of respiration. These results show that although part of the observed decrease in ATP in the treated cells at high Synkavit concentrations may be due to an inhibition of respiration, at low concentrations where the oxygen uptake is unaffected or slightly stimulated, there is still a considerable decrease in ATP in the cells.

*Possible effect of Synkavit on the assay procedure*

A standard solution containing 20 $\mu$g. ATP in 0·1 ml. was assayed six times. For the first two assays distilled water was added to bring the volume up to

TABLE III.—*Effects of Synkavit on Endogenous Respiration of Ascites Tumour Cells*

| Experiment | M conc. Synkavit | μl/hr/ml. ascites fluid | | % Change | |
|---|---|---|---|---|---|
| | | First 5 mins. | Half-hour | Initial | Half-hour |
| 1 | .. | 204 | 126 | .. | .. |
| | $3 \times 10^{-2}$ | 168 | 106 | −18 | −16 |
| | $3 \times 10^{-4}$ | 240 | 134 | +18 | +6 |
| *2 | .. | .. | 42·5 | .. | .. |
| | $3 \times 10^{-2}$ | .. | 17·5 | .. | −59 |
| | $3 \times 10^{-4}$ | .. | 46 | .. | +8 |
| 3 | .. | 216 | 151 | .. | .. |
| | $3 \times 10^{-2}$ | 174 | 107 | −19 | −29 |
| 4 | .. | 456 | 310 | .. | .. |
| | $3 \times 10^{-4}$ | 588 | 313 | +29 | +1 |
| 5 | .. | 180 | 447 | .. | .. |
| | $3 \times 10^{-6}$ | 186 | 429 | +3 | −4 |
| 6 | .. | 222 | 222 | .. | .. |
| | $1 \cdot 5 \times 10^{-3}$ | 183 | 183 | −17·5 | −17 |

* Tumour was 14 days old.

0·6 ml. In the other assays solutions of Synkavit were added to bring the Synkavit concentration to $1 \times 10^{-2}$ M and $1 \times 10^{-4}$ M.

The changes in optical density in 5 minutes were as follows :

| | |
|---|---|
| ATP alone | 0·079 ± 6 per cent |
| ATP + $1 \times 10^{-2}$ M Synkavit | 0·0495 ± 1 per cent |
| ATP + $1 \times 10^{-4}$ M Synkavit | 0·072 ± 3 per cent |

Thus at the highest concentrations of Synkavit added, as much as 36 per cent of the observed decrease in ATP may be due to an interference with the assay procedure. This value is probably a maximum however, since the Synkavit has almost certainly beeen altered chemically at the end of half an hour. Material fixed to proteins or changed to a quinone is not extracted by the trichloracetic acid (Chipperfield and Marrian, unpublished) so that the concentration of Synkavit in the extracts is probably very low. At the lower concentration of Synkavit the possible error in the assay is not significant.

It has been suggested that quinol phosphates could be involved in phosphorylation processes accompanying the passage of electrons from substrates to oxygen inside living cells (Clark, Kirby and Todd, 1958 ; Harrison 1958). Clark *et al.* showed that if Synkavit were oxidised in the presence of inorganic phosphate, pyrophosphate was formed, and in a later paper (Clark, Hutchinson and Todd, 1960) that ADP could similarly be produced from AMP. More recently, Chmielewska (1960) and Dallam (1961) have suggested mechanisms for oxidative phosphorylation in which the presence of an isoprene unit on position 3 of the quinone molecule is necessary for phosphorylation to take place. A quinone which did not have such a substituent could act as a carrier of electrons, but could not be a coenzyme for oxidative phosphorylation. Recent support for these theories has come from the work in bacteria by Russell and Brodie (1961) who showed that β-chroman derivatives of Vitamin $K_1$ and analogous compounds could be detected in bacterial preparations which were capable of oxidative phosphorylation. Only compounds which were capable of forming this chroman ring were active in restoring phosphorylation to ultra-violet-irradiated bacterial preparations. Furthermore, it has been shown that liver mitochondria from X-irradiated animals had a reduced capacity for oxidative phosphorylation and that pre-treatment with either Vitamin $K_1$ or Vitamin E lessened the effect (Nitz-Litzow and Bührer, 1960). If Synkavit is dephosphorylated inside the ascites tumour cell, the menadione produced could take part in the transfer of electrons from substrates to oxygen, but not in the accompanying phosphorylation processes. Our results could therefore be due to the menadione taking part in the electron transport processes of the ascites cell in place of Co-enzyme $Q_{10}$ or some related natural quinone. This electron transport could not produce ATP, so the net result would be a decrease in ATP in the treated cells, the cell respiration being essentially unaltered. However, the observed decrease in ATP in the cell might also be due to a stimulatiod of the hexose monophosphate pathway of metabolism. Wenner, Hackney and Moliterno (1958) showed that menadione and other compounds stimulated the metabolism of glucose along this pathway in ascites tumour cells and it has since been shown (Hoskin, 1960) that Synkavit as well as menadione has a stimulatory effect on the hexose monophosphate shunt in brain tissue, which resembles tumour tissue in having a low concentration of NAD. This stimulation could be due to the menadione preferentially oxidising $NADPH_2$, which is produced by the enzymes oxidising glucose-6-phosphate. This would divert the glucose-6-phosphate and cause the striking inhibition of aerobic glycolysis observed by Tiedemann *et al.* (1958) in ascites tumour cells.

Similar work on other quinones suggests, however, that the decrease in ATP and the effect on glycolysis may be secondary effects due to an effect on NAD. Tiedemann and Risse (1960) studying the effects of 9:10-phenanthraquinone and a water-soluble derivative on ascites tumours showed that this quinone also inhibited glycolysis. There was a marked reduction in ATP and a sharp fall in the level of NAD. In the absence of glucose the only effect observed was a fall in the NAD content, suggesting that the effect on NAD was the primary one. Since Quagliariello *et al.* (1959) have claimed that vitamins $K_1$ and $K_2$, menadione and Synkavit inhibit the synthesis of nicotinic acid, a decrease in NAD could be the cause of the decrease in ATP and inhibition of glycolysis.

### SUMMARY

The radiosensitising agent, Synkavit, reduces the ATP content of Ehrlich mouse ascites tumour cells *in vivo* at dose levels of about $1\mu$ Mole/gram and *in vitro* at concentrations between $3 \times 10^{-2}$ and $3 \times 10^{-6}$ molar. The higher concentration of the substance reduced the respiration, while the low concentration caused a small stimulation *in vitro*.

The authors wish to thank Professor J. S. Mitchell, F.R.S., for his interest and advice and the Medical Research Council for financial assistance to one of them (B.C.).

### REFERENCES

CHMIELEWSKA, I.—(1960) *Biochim. biophys. Acta*, **39**, 170.
CLARK, V. M., HUTCHINSON, D. W. AND TODD, A. R.—(1960) *Nature*, **187**, 59.
*Idem*, KIRBY, G. W. AND TODD, A. R.—(1958) *Ibid.*, **181**, 1650.
DALLAM, R. D.—(1961) *Biochem. Biophys. Res. Commun.*, **4**, 106.

DARCIS, L. AND CLOSON, J.—(1959) *Excerpta med., Amst. Sec. XIV*, **13**, 57, 254.
HANEL, H. K., HJORT, G. AND PURSER, P. R.—(1958) *Acta Radiol.*, **49**, 401.
HARRISON, K.—(1958) *Nature*, **181**, 1131.
HOSKIN, F. C. G.—(1960) *Arch. Biochem.*, **91**, 43.
MARRIAN, D. H.—(1959) *Brit. J. Cancer*, **13**, 461.
MITCHELL, J. S. AND SIMON-REUSS, I.—(1952) *Ibid.*, **6**, 305.
NITZ-LITZOW, D. AND BÜHRER, G.—(1960) *Strahlentherapie*, **113**, 201.
QUAGLIARIELLO, E., SACCONE, C., RINALDI, E. AND ALIOTO, M. R.—(1959), *Nature*, **184**, 820.
RUSSELL, P. J. AND BRODIE, A. F.—(1961) ' Quinones in Electron Transport '. London (Churchill).
STEIN, J. A. AND GRIMM, M. L.—(1959) *Nature*, **182**, 1681.
STRENGTH, D. R. AND SEIBERT, M. A.—(1955) *Proc. nat. Acad. Sci., Wash.*, **41**, 609.
TIEDEMANN, H. AND RISSE, H. J.—(1960) *Experientia*, **16**, 319.
*Iidem* and BORN, J.—(1958) *Z. Naturf.*, **13b**, 657.
WATTENBERG, L. W.—(1961) ' Quinones in Electron Transport '. London (Churchill), p. 367.
WENNER, C. E., HACKNEY, J. AND MOLITERNO, J. H.—(1958) *Cancer Res.*, **18**, 1105.